# Alimentation

**Published:** 1882-08

**Authors:** J. F. Sanborn

**Affiliations:** Tabor, Iowa


### ALIMENTATION.

BY J. F. SANBORN, D.D.D.S., OF TABOR, IOWA.

[Read before the Iowa State Dental Society.]

Alimentation, in a physiological sense, is the chief end of man's physical, vital action in the developing of brain and nerve force, or the power to think and act.

Charles Lamb was once asked, "What is matter?" answered "Never mind." "And what is mind?" answered "No matter." From his answers, though true, we can form but a very imperfect idea of either mind or matter, or their relations to each other. It is true that mind is not matter, but what knowledge of mind can we obtain without observing it in its relationship with matter, and without mind we can form no conception of matter. Without brain and nerve force, no vital action can take place. No respiration; no circulation; no muscular action—nothing but a dead, inert, corporality; but, with a harmonious activity of the brain and nerve force, ruling and directing all vital actions, and we have the full, free, active condition that constitutes life in the highest acceptation of the term. Physical life, or the actions that support life, are dependent on nerve and brain force for their continued activity. Life, then, is an active condition of the vital changes that support brain and nerve force, the acknowledged medium of mentality. Health is the perfect balance in the vital changes. The cause of disease is the loss of balance in these changes. Disease is nature's effort to restore this loss of balance; and death is the cessation of these vital changes. Physiology teaches the normal action of all the organs of vitality.

It is the duty of every physician to thoroughly acquaint himself with physiological conditions, so that whenever the balance is lost in any of the vital actions, he may intelligently know how to aid and assist the "vis medicatrix naturæ" in restoring the normal balance, and convalescence is sure to follow. This is an important duty and requires great research, careful thought, a sound discriminating judgment, courage to perform, and when the task is well executed, the physician's responsibility terminates. These vital actions may be divided into two classes, viz.: the build up, and the take down of tissue, each of which are again divided into two, making four in all.

1st. Alimentation, which embraces all the changes from food received till it becomes Plasma in the blood.

2d. Assimilation, which embraces all the changes of plasma in the blood till it becomes the various tissues of the body. These are the build up actions.

3d. Disintegration embracing all the changes incident to the oxidation, or break down of tissue.

4th. Depuration or the removal of broken down tissue.

Your attention is now invited to the first class of vital changes. alimentation, aliment strictly and physiologically speaking implies, that which affords nourishment, and embraces such food as are demanded to build up the tissues; anything other than this is strictly speaking, condiments, or poisons; a something to please the taste, or to gratify an abnormal appetite, and tends rather to destroy the beautiful, harmonious balance in the vital changes, that constitute perfect health; and are to a greater or less extent the primary cause of disease. There are four classes of foods that are digested, to nourish the various tissues.

1st. The proteids, or albumenous foods, are composed of $C_{48}$, $H_{36}$, $O_{14}$, $N_6$ plus atrace of S. This class of foods are known as Nitrogenous because they contain N. in contradistinction to those that have no nitrogen. Fibrine, muscular tissue, gluten, colloids, casein of milk, the white of the egg, are of this class, and are called protids, or albumenoids; and when digested become albuminous or peptose, and are not coagulated by heat as are fibrine and albumen. These various foods when introduced into the blood, after being duly digested, are the part of the plasma used by the bioplasts to form muscular tissues, and its congeners. The second class are called Amylaceous, which means starchlike, and is composed of $C_{12}$, $H_{20}$, $O_{10}$, and in all of them the H. and O., are in the proportion that they form water, and embrace starch, dextrine, liquin, cellulose, cane sugar, milk sugar, and the sugar of fruits. Glycogen is the animal starch, and is of the same chemical atoms as the molecules and the same number, as vegetable starch.

Lignin or wood fiber is also of the same chemistry as starch, but the digestive organs of man are not capable of dissolving it, so it is not an article of importance except for ruminating animals. The third class are fats and oils, and are chemically composed of $C_{12}$, $H_{32}$, $O_{12}$, in which the H. and O. are not in the proportion to form water. The fats are composed of stearine, margarine and oleine. The greater the amount of the stearine in proportion to the other ingredients, the harder will be the fat, and the greater the amount of oleine in proportion to the other ingredients, the softer it will be; so that the chemical formula is but an average of the various kinds of oil and fat.

As the H. is the lightest of all the gases, and as it is greatly in excess of the O, in the fats and oils, as compared with what it is found in water, the specific gravity of oils are less, and float on it, and they have such a repugnance to uniting with water, that it always becomes necessary to call a third ingredient to cause them to unite. The fourth class of foods is that which nourishes the osseous structure which is composed of inorganic elements, so called, 66 p. c. and 34 p. c. of organic matter. The inorganic elements so called are just as truly organic, and the result of growth, as any of the other tissues.

The lime salts of the eggshell, or of the bones of an animal are as truly organized from the plasma of the blood made from the grains used as food, as are any of the other tissues, and the idea that fowls eat lime mortar of old walls, to furnish the wherewith to make shells for the eggs they lay, has long since passed to be numbered among the theories founded on the ignorance of the past. The organic bone materials are developed from that part of our food that becomes ashes when consumed, and are just as truly organic, as the starches, sugars or gluten, that nourishes the soft tissues. The idea that our tissues are all changed once in seven years, has long since been numbered among the things of the past.

The osseous structure is the frame work for the use of the other parts of the body and the metabolism is vastly slower than that of the soft structures, and are probably all replaced at least once a year while the soft structures are changed several times every twelve months. In fact the most marked characteristic of animal life is its unceasing change. Digestion is the rendering soluble the various foods eaten.

As has been shown the albumenoids, amylaceous or carbohydrates and oils are so different in their structure, it is obvious that a different menstrum must be used to render each into a fluid condition.

Aside from the inorganic elements just noticed, there are three classes of aliment and three digestive fluids, and three different locations where digestion takes place.

The first of these menstrums is the gastric juice which has an acid reaction. The succus entericus is alkaline, the bile and pancreatic juice are alkaline. The acids and alkalies bear the relationship of electro postive and negative to each other.

Unlike electric conditions attract each other while like conditions repel. The gastric juice being acid in its reaction, and the saliva alkaline, the food if well moistened with the saliva, will the more readily mix with the gastric juice than if moistened with water, tea, coffee, wine, or any other liquid. This accounts for one cause of dyspepsia; haste in eating, swallowing the food before it has become insalivated and in order to become sufficiently moist to swallow has been moistened with water, tea, coffee, etc,, which are electro neutral, so the gastric juice does not become readily mixed with it; fermentation the first process in decay, takes place instead of digestion, an acid is formed by the fermentive process; this acid and the acid of the gastric juice repel each other, and indigestion is the consequence.

The idea that digestion is a fermentive process, has not the shadow of foundation in fact. Fermentation is a break-down action, a decomposing of original molecules, and the formation of new compounds; digestion is a dissolving, or rendering of a solid or semi solid into a fluid, without changing the relationship of the original ultimate atoms of the molecule and without the re-formation of new compounds. In the change of starch and dextrine to dextrose there is what physiologists call a hydration, the addition of H. and O. in proportion that forms water, but there is no decomposing of the original molecules, and a re-forming of the atoms to make new compounds, as is the case when fermentation takes place.

The albumenoids by the action of the pepsin of the gastric juice, are changed to albumenose or peptose as sometimes called, which is the soluble elements of muscular tissue, and is in condition to be absorbed into the blood as plasma for the bioplasts to absorb and use.

The stomach has four coats, first, the peritoneal; second, muscular; third, vascular; fourth, mucus. The mucus coat is abundantly supplied with follicles and depressions.

These follicles are abundantly supplied with veins and arteries from the vascular coat. The follicle has a gland at the bottom, that secretes the gastric juice, this gland extends about half way up the follicle and the balance of it is a duct lined with epithelium. These glands secrete the digestive fluids. The inter-follicular space is in its anatomy very much like the villi of the small bowels. It has an artery that supplies the gland, and a vein plexus to absorb the disgestive albumenose.

It is through these gastric villi that the digested contents of the stomach find their way into the circulation. The albumenoids are muscular tissue, cellular capsule of the fat, caseine of milk, gluten of the cereals, and the like. If the digestion of these albumenoids is but imperfectly performed the pancreatic juice is said to complete it and the veins of the villi absorb it.

The second class of foods, the amylaceous, embrace starch, dextrine, cane sugar, glucose, the sugar of milk and the sugar of fruits. These are not acted on by the gastric juice, but pass the pyloric orifice to the duodenum where Brunner's glands furnish the succus entericus, that digests them; changing the starch and dextrine by the addition of H. and O. in the proportion that forms water two parts and to cane sugar one part, and they become dextrose.

The sugar of fruit is grape sugar, and of the same chemistry as glucose and dextrose, $C_{12}$, $H_{24}$, $O_{12}$.

These amylaceous foods when digested are called dextrose, and are absorbed by the veins of the villi, and pass by the portal system to the liver.

Brunner's glands are situated in the duodenum and somewhat resemble in their structure, the salivary glands, and furnish an alkaline digestive fluid known as succus entericus. As the contents of the stomach are acid when they leave it, they become more readily mixed with the intestinal juices, because it is alkaline. Glucose means sweet, and was formerly applied to the sugar of grapes and other fruits; but to-day there is a saccharine article placed on the market that is manufactured from starch, by boiling it with a diluted acid, and afterward neutralize the acid with an alkali, usually a hydrate of lime; a portion of it will crystalize, and when claritied has the appearance of cane sugar; but has only a small part of sweetening properties. It is used to adulterate the cheap grades of cane sugar, and for the manufacture of candy. The part of glucose that does not crystalize, is sold as silver drips, golden drips and the like, and have but small sweetening properties when compared with cane syrup.

Glucose has the same chemical constituents as grape sugar; but in its manufacture the change brought about is very different from the change produced by growth in the vegetable. The starch as found in the cereals has life giving properties, and does nourish and build up tissue; and so does the grape, sugar of grapes and other fruits; but when glucose is made by chemical action, the nourishing life-giving properties are entirely lost, as has been demonstrated by feeding it to bees.

The chemistry of glucose is the same as honey, the bees will thrive on honey, but languish and die if confined to a diet of glucose. The bee will live and thrive on dissolved cane sugar, or cane syrup, but die when confined to the artificial imitation, which man with all his superior intelligence uses without receiving any benefit, and pays his money for that which does not satisfy.

In the digestive process, the grape sugar is not changed by hydration as are starch and cane sugar, and for this reason in part, the fruits as apples and grapes, are so perfectly adapted to the wants of the body, as compared with the cane sugar purchased in the market and used to add to the sweets of life.

Glucose is artificially made from starch; dextrose is digested starch, dextrine and cane sugar. Grape sugar is the sugar of the grape and other fruits. Dextrose, when it is absorbed by the villi passes to the liver, by the portal vein, where the bile is removed from it, and it then passes by the hepatic vein into the vena cava and into the heart and lungs where it is further changed to the elements of fat, from which the bioplasts of the adipose tissue change it to fat.

The books on physiology teach us that dextrose is found in the right side of the heart, but that no trace of sugar can be found in the blood of the left side. This will be better understood by seeing what change is necessary to convert dextrose into fat. Dextrose is composed of $C_{12}$, $H_{24}$, $O_{12}$, and fat is $C_{12}$, $H_{32}$, $O_{32}$. Now by the addition of $H_8$ to the dextrose, and we have the same chemistry as fat.

Just where this $H_8$ comes from has never been demonstrated, but where molecular change is so abundantly taking place as it does in the lungs, there are ample sources for it.

During digestion the flow of dextrose to the liver is superabundant and much too fast for this metabolis to take place in the lungs.

After digestion is completed, the supply would be less than the demands of vitality require, so to compensate for this unequal flow of dextrose into the portal circulation, the liver performs the office of a balance wheel by changing a portion of the dextrose to animal starch, or glycogen and stores it up, while the flow is superabundant and again changes glycogen to dextrose when the flow is less than the vital demands require.

Whenever the liver becomes unable to preserve this beautiful balance, dextrose is passed to the lungs faster than the normal change can take place, then a portion of it only becomes the elements of fat and the balance passes into the circulation as dextrose, or glucose, as it is more commonly called. As this glucose is an abnormal constituent of arterial blood, the kidneys remove it with the urine, and if this abnormal condition becomes chronic we have the disease known as mellitus diabetes, which is primarily, most emphatically, a disease of the liver, and not of the kidneys.

The liver then performs the double duty of removing the bile from the blood of the portal system, and regulates the flow of dextrose to the lungs. If from any cause the liver neglects to perform the first of these duties, biliousness in some of its forms is the result, and if it omits to perform the second, for any very considerable time, mellitus diabetes is the result.

In the treatment of these diseases due consideration should be had to the lesion of the liver as the primary cause; while the kidneys may become involved secondarily, in attempting to remove the glucose from the blood. The treatment is very difficult, so long as the liver lesion continues. Mellitus diabetes has been considered a kidney disease, and its treatment has baffled the skill of the most learned physicians for generations.

It is hardly necessary to state that albuminuria is an entirely different disease from mellitus diabetes. As the kidneys do vicarious duty for the liver, as has been just shown, so the lungs may, and often do perform vicarious duty for the liver; and in so doing, have sometimes become involved in a modified phthisis pulmonalis, which, if it has not progressed too far, may be cured by causing the liver to resume its bile secreting function, by such treatment as shall remove the hepatic lesion. The lungs on being relieved of their extra duty, have resumed their normal action, and the physician may claim high honors for curing tuberculosis of the lungs, when he has only removed the hepatic lesion; which shows his ignorance in diagnosis, instead of skill in therapeutics.

The third class of aliments are the fats and oils, either animal or vegetable. They are usually found inclosed in a covering of proteine, and when eaten, this capsule is digested in the stomach by the gastric juice, and the fat is set free, but not acted on, and passes on unchanged until past the meatus of the ductus communis choledochus, then it becomes mixed with the bile and pancreatic juice. This mixed fluid is alkaline in its reaction.

All authors have agreed that the bile is the principal agent employed in the digestion of fats and oils, but they are not all agreed as to the pancreatic juice rendering it any assistance.

Foster claims that the chief functions of the trypsin is to finish up the digestion of the parapeptones left undigested by the pepsin, as they are both alkaline in their reaction, they probably act in harmony in doing whatever comes within their province to do for the general good.

After the fats have been emulsified, they are absorbed by the lacteals of the villi, pass by the way of the mesenteric glands to the thoracic duct and are found as chyle, mixed with lymph from the lymphatic system.

We eat and drink primarily for the purpose of furnishing pabulum for the bioplasts to build up and repair waste tissues that are constantly becoming necessary as a condition of life and health.

In the break down of tissue, a certain portion of the disintegrated matter is carbonic dioxide. A portion is a solid or semi-solid as fæces, and a third portion is a fluid surcharged with such solids as may be dissolved in it. This fluid part is absorbed by the lymphatics, which are more numerous, and everywhere accompany the veins. The lymphatics of the upper right side, arm and head, pour their contents into the right subclavian vein, the lymphatics of the balance of the body, left arm and head, pour their contents into the thoracic duct in common with the emulsified fat. The mixture of the two forms the chyle, a milky-like fluid, which enters the circulation through the left subclavian vein.

This lymph, as found in the lymphatic vessels and glands, is the liquid portion of the broken down tissues of the whole body, and constitutes the larger portion of the urea which is strained out of the blood by the kidneys. If this lymph is not absorbed and removed by the lymphatics, but remains in the tissues where it is formed by the breaking down process, it becomes the accumulated fluid that constitutes the disease known as dropsy.

Now, if the fat passes into the circulation thoroughly mixed with this lymph, what relationship as an aliment does it bear to the physiological wants of vitality? That the lymph has its origin from the disintegration of tissue, and more especially of the proteids, we have but to look at what chemical changes would have to take place to accomplish this end.

Muscle tissue is chemically composed of $C_{48}$, $H_{36}$, $O_{14}$, $N_6$. The tissues are disintegrated by the oxygen we breath for that sole purpose. The N. united with $H_{18}$, $N_6$ equals 6 $(NH_3)$ or six parts of ammonia.

The other $H_{18}$, united with $O_9$, would form 9 $(H_2O)$. or nine parts of water. We now have $O_5$, and $C_{48}$, left by the addition of $O_{91}$ to the $O_5$, we have $O_{96}$, which will unite with $C_{48}$, to form 48 $(CO_2)$, or forty-eight parts of carbonic dioxide.

The carbonic acid gas passes by the venous blood to the lungs, and is there expelled. The ammonia unites with the water to form urine, and is removed from the blood by the kidneys.

Now if the fat passes into the blood thoroughly mixed with this lymph, what relationship as an aliment does it bear to the physiological wants of vitality? When cooked into food, as in frying meat and potatoes, or used as shortening in pastry it has to be removed from the pabulum before digestion can take place. It is digested by the action of the bile, which is an excretory fluid removed from the blood of the portal system by the liver for this purpose the better to prepare the blood for the circulation, and if not removed is a cause of disease.

After the fat is digested it passes into the circulation, as has been shown, mixed with the lymph, a refuse dead matter, on its way out of the system; it is removed from the blood by being strained out by the tissues, and not formed by normal bioplastic action, as is every other tissue of the body; and when oxidized for removal, it is done like any other dead matter, and removed by the organs of depuration as carbonic acid gas and water. Is the abundant use of animal fat mixed with and constituting a portion of our three times a day indulgence, clogging the system, and preventing its free, full, healthy activity, so essential to the highest and most enjoyable living?

The physiological demands of vitality for the aliment necessary to maintain its most vigorous activity and usefulness is worthy of your highest consideration; and the practical daily conformity of our habits should be governed by these laws, rather than the caprice of an indulged appetite. In the treatment of the sick these principles should not only not be ignored, but should guide the physician in selecting and prescribing for his patient what he should, as well as what he should not eat. In acute disease the vital action is not in building up tissue, but rather in restoring the lost balance in vital action. During such a condition the practice is most emphatically reprehensible of urging the patient to take food when the appetite loathes it; the plasma in the blood is already overloaded with that which cannot be used, and thereby increase rather than diminish the complication of evils that vitality has to contend with. Let us always remember that the highest attainments in health and usefulness are attained only by strict obedience to physiological law.

